# The Effects of Different Induction Chemotherapy Cycles and Adjuvant Chemotherapy on the Survival Outcomes of Patients With Locally Advanced Nasopharyngeal Carcinoma

**DOI:** 10.3389/fonc.2022.845704

**Published:** 2022-06-20

**Authors:** Shu Liao, Yunlian Diao, Qingyuan Ling, Zhijuan Xiong, Wenxin Deng, Ping Zhang, Congkai Zhang, Ying Ying, Xiaojun Zhong, Wei Zhang

**Affiliations:** ^1^ The Department of Respiratory and Critical Care Medicine, The First Affiliated Hospital of Nanchang University, Nanchang, China; ^2^ The Department of Oncology, The First Affiliated Hospital of Nanchang University, Nanchang, China; ^3^ Jiangxi Institute of Respiratory Disease, The First Affiliated Hospital of Nanchang University, Nanchang, China; ^4^ Human Genetic Resources Center, The First Affiliated Hospital of Nanchang University, Nanchang, China; ^5^ Jiangxi Medical Center for Major Public Health Events, The First Affiliated Hospital of Nanchang University, Nanchang, China

**Keywords:** locoregionally advanced nasopharyngeal carcinoma, induction chemotherapy, adjuvant chemotherapy, cycle numbers, survival

## Abstract

**Objective:**

This study investigated whether differences in the induction chemotherapy (IC) cycle number and adjuvant chemotherapy (AC) affect survival outcomes in patients with locally advanced nasopharyngeal carcinoma (LA-NPC).

**Methods:**

The survival outcomes of 386 consecutive LA-NPC patients treated between January 2015 and March 2018 were retrospectively analyzed. Univariate and multivariate analyses were used to compare treatment groups defined by IC< 3 or ≥3 IC cycles followed by radiotherapy with or without AC (i.e., IC<3+AC, IC<3+non-AC, IC≥3+AC, and IC≥3+non-AC groups).

**Results:**

The median follow-up time was 53 months (range: 2-74 months) and the median number of IC cycles was 2 (range: 1-6 cycles). The 3-year overall survival (OS) rate was significantly higher in patients with IC≥3 cycles compared to IC<3 cycles (95.7% vs. 90.3%, *P*=0.020). Multivariate analysis indicated that the IC cycle number is an independent factor for OS (hazard ratio=0.326, *P*=0.007). Furthermore, patients in the IC<3+AC group had a better OS rate than those in the IC<3+non-AC group (91.6% vs. 79.1%, *P*=0.030), indicating that AC positively affected OS in patients with IC<3. However, no significant difference in the OS rate was found between IC≥3+non-AC and IC≥3+AC groups (92.1% vs. 94.6%, *P* =0.550).

**Conclusion:**

The IC cycle number appears to be an independent prognostic factor for higher OS in LA-NPC patients who received ≥3 cycles. Sequential AC after IC plus radiotherapy may improve OS in patients with IC<3 cycles.

## Introduction

Nasopharyngeal carcinoma (NPC), arising from the nasopharynx epithelium, is a type of head and neck cancer that is especially common in Southern China (1). In clinics, 70% of patients with NPC are diagnosed with a locoregionally advanced (LA) stage (2, 3). Due to the complexity of the anatomical location and the high radiosensitivity of this cancer type, radiotherapy (RT) is considered the most effective treatment strategy for NPC. However, RT alone may not be sufficiently effective to eliminate locally advanced nasopharyngeal carcinoma (LA-NPC), and thus, intensity-modulated radiotherapy (IMRT) and concurrent chemoradiotherapy (CCRT) are widely used as the primary treatment options for LA-NPC. To further increase the effects of CCRT/IMRT, several other intensified therapeutic strategies have been recently developed as supplemental treatments, such as induction chemotherapy (IC) or adjuvant chemotherapy (AC).

A growing body of evidence suggests that IC can provide distinct improvements to LA-NPC patient outcomes, for example, through earlier elimination of distant micro-metastasis and by producing higher patient tolerance to subsequent radiotherapies (1, 4–7). A phase II clinical study has shown that IC followed by CCRT can significantly increase the overall survival rate (OS) of LA-NPC patients compared with CCRT alone (4). Subsequent large-scale, multi-center, phase III clinical trials further showed that IC-combined CCRT can improve progression-free survival (PFS) and OS and decrease the occurrence of distant metastasis (5–7). A pooled analysis of randomized experimental data from four epidemic areas found that IC followed by CCRT can improve 5-year OS by increasing distant metastasis-free survival (DMFS) (8). These cumulative findings confirmed that IC followed by CCRT/IMRT can serve as a promising option for patients with LA-NPC.

However, the optimum number of IC cycles for LA-NPC patients remains unclear. Previous studies have most commonly tested the effects of 2–4 cycles of IC in NPC patients (4, 5, 9–12). In addition, two recent propensity score studies indicated that more than 2 cycles of IC provided no further benefits to patient survival (13, 14). However, another study of 1,164 patients with N2-3 NPC who received 0, 2, or 4 cycles of IC showed that 4 IC cycles could lead to improved survival endpoints (e.g., OS, DMFS, locoregional relapse-free survival (LRFS), and PFS) (15), indicating that the administration of IC cycles warranted further exploration and optimization.

Additionally, some LA-NPC patients have been treated with a combination of IC followed by CCRT/IMRT plus AC in clinical practice. For instance, He et al. found that IC treatment followed by IMRT plus AC is effective and safe in LA-NPC patients (16). By contrast, Zou et al. reported that sequential AC following IC+CCRT provided no additional benefits to patient survival, and, in fact, resulted in a higher incidence of acute toxicity (17). Thus, it remains unclear whether IC followed by CCRT plus AC can improve outcomes among LA-NPC patients.

The objective of this study was to further investigate the optimal cycle numbers of IC cycles and to test whether supplemental AC treatment could enhance the efficacy of IC plus CCRT/IMRT for LA-NPC. We retrospectively investigated 386 patients with consecutive LA-NPC who received treatment with IC followed by CCRT/IMRT with or without AC, and analyzed the impacts of various IC cycles as well as the effects of AC, on survival outcomes among LA-NPC patients.

## Materials and Methods

### Patients

We retrospectively analyzed the data on patients with pathological and newly diagnosed NPC in the First Affiliated Hospital of Nanchang University from January 2015 to March 2018. The inclusion criteria were as follows (1): the diagnoses of stage III-IVA according to the 8th edition of the Union for International Cancer Control/American Joint Committee on Cancer (UICC/AJCC) staging system; (2) scores ≥ 80 in the Karnofsky scale and were between ages 18 and 70; (3) received at least one cycle of IC prior to IMRT; (4) completed the treatment plan with complete clinical information and follow-up data; (5) exhibited normal heart, lung, liver, and renal function, without other cancers or pregnancy.

**Figure 1:**
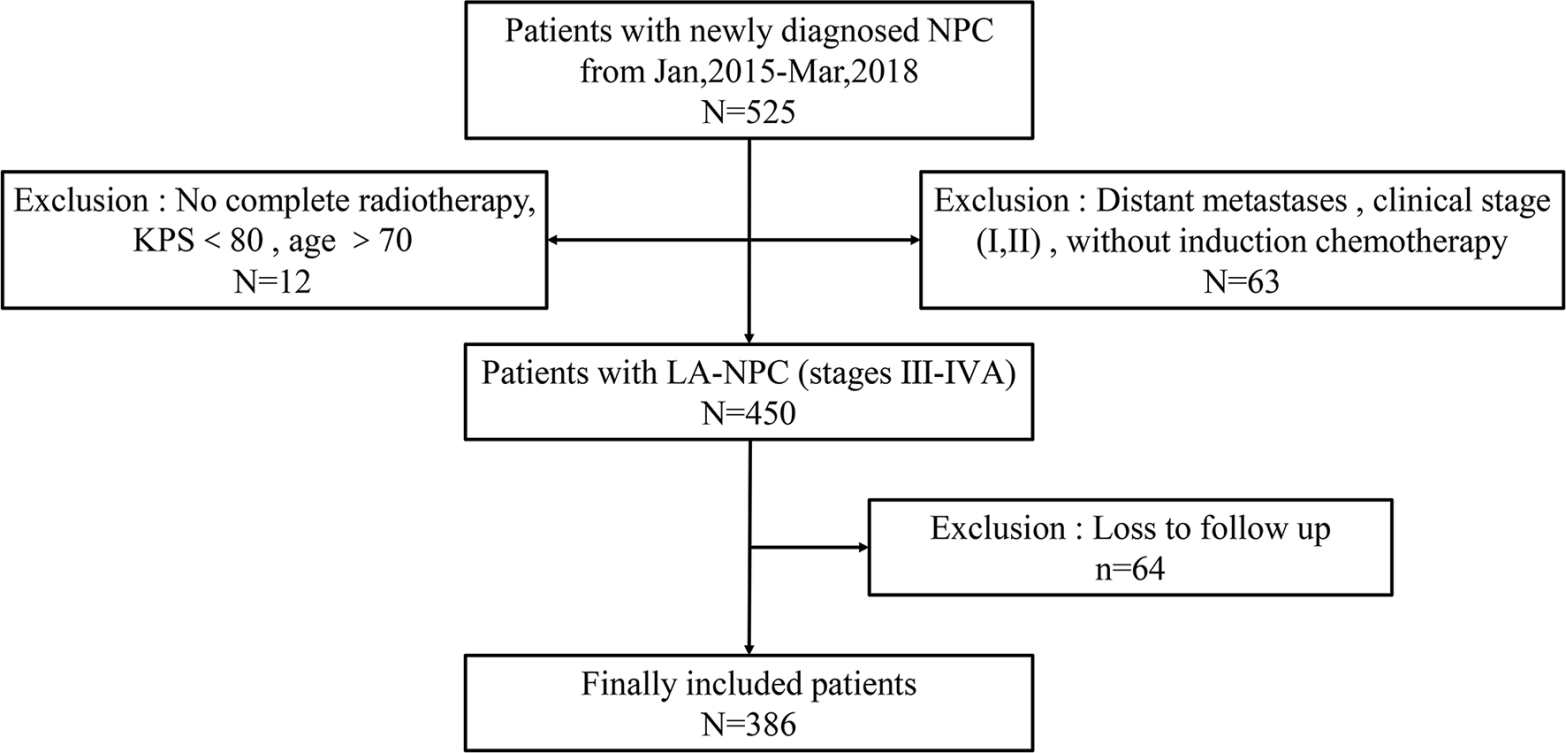
Schematic for patient selection and inclusion in this study. NPC, nasopharyngeal carcinoma; LA-NPC, locally advanced nasopharyngeal carcinoma; KPS, Karnofsky scale.

Based on these criteria, a total of 386 LA-NPC patients were selected for the study. The entire patient enrollment procedure is summarized in **Figure 1**.

### Pre-Treatment Examination

For all patients, pathological diagnoses were obtained by direct fiberoptic nasopharyngoscopy. According to the 2003 edition of the World Health Organization (WHO), pathological classifications were based on the following criteria: type I includes keratinizing squamous cell carcinoma; type IIA is non-keratinizing differentiated carcinoma and type IIB is non-keratinizing undifferentiated carcinoma; and type III is basal cell-like squamous cell carcinoma. The clinical stage was evaluated according to the eighth edition of AJCC/UICC, and the workup included the magnetic resonance imaging (MRI) of the head and neck, chest radiography (DR) or chest CT, abdominal ultrasound (B-US) or CT, whole-body bone scan (ECTs), and/or whole-body PET-CT examination.

### Radiotherapy

All patients received one fraction of IMRT (6 MV photons) daily for 5 days each week. The primary nasopharyngeal gross tumor volume (GTVnx) and the involved cervical lymph nodes were determined based on MRI/CT and/or PET-CT imaging, clinical evaluation, and endoscopic findings. The target volumes were delineated following reports 50 and 62 by the International Commission on Radiation Units and Measurements. The prescribed radiation dose is defined as follows: 68–72 Gy to the planning target volume (PTV) of the GTVnx (including the primary tumor and enlarged lymph nodes), 64–70 Gy to the PTV of the nodal gross tumor volume (GTVnd; including enlarged lymph nodes in the neck), 60–63 Gy to the PTV of CTV1 (high-risk regions), and 54–60 Gy to PTV of CTV2 (low-risk regions) in 30–33 fractions.

GTVnx and the neck metastatic lymph node (GTVnd) were delineated based on the pre-treatment MRI or CT of the head and neck. CTV1 was delineated to cover sites with a high-risk of microscopic spread (including GTVnx with a margin of 5–10 mm). CTV2 was delineated to cover the low-risk sites of microscopic extension (including CTV1 with a margin of 5–10 mm) and potentially involved regions and lymphatic regions. After trimming, CTV1 or CTV2 can only be expanded by 2-3 mm adjacent to the brainstem and spinal cord.

### Chemotherapy

In this study, all patients received at least 1 cycle of IC before radiotherapy. The regimens of AC and IC were same and included the following: TP (paclitaxel 135 mg/m^2^ on the first day and cisplatin/nedaplatin 25 mg/m^2^/day on the first 3 days), DP (docetaxel 75 mg/m^2^ on the first day and cisplatin/nedaplatin 25 mg/m^2^/day on the first 3 days), and TPF (cisplatin/docetaxel 60 mg/m^2^ on the first day and fluorouracil 600 mg/m^2^/day as a continuous 120 h infusion on days 1–5), which were administered every 3 weeks. Concurrent chemotherapy (CCT) regimens consisted of a single-drug platinum or fluorouracil weekly for a maximum of seven cycles.

### Endpoints and Toxicity

Follow-up was measured from the day of initial pathological diagnosis to last examination or the date of any endpoint event. Patients were assessed every 3 months during the first 2 years, then every 6 months thereafter, or until death. The follow-up endpoints were the OS rate, locoregional relapse-free survival (LRFS) rate, DMFS rate, and PFS rate.

Toxic reactions included blood toxicity (leukopenia, neutropenia, anemia, thrombocytopenia) and adverse events listed under the National Cancer Institute Common Terminology Criteria for Adverse Events NCI-CTCAE ver. 4.0.

### Research Group

All patients received IC for 1-6 cycles before radiotherapy. Since the median number of IC cycles was 2 cycles, patients given <3 cycles of IC were referred to as the IC<3 group, while patients administered with ≥3 cycles of IC were referred to as the IC≥3 group.

Among the total cohort, 224 (58%) received adjuvant therapy (AC) sequentially after CCRT/IMRT. Since patients in the IC<3 group received AC significantly more frequently than those in the IC≥3 group (68.4% vs. 39.6%, *P*<0.001), we therefore subdivided the patients into four treatment groups, that is, IC<3+AC, IC<3+non-AC, IC≥3+AC, and IC≥3+non-AC.

### Statistical Analysis

Continuous variables were analyzed by the T-test, and categorical variables were analyzed by the chi-square test. The Kaplan–Meier survival function was used to compare survival rates, draw survival curves, and perform log-rank tests of the survival rate for each group. Single and multivariate Cox regression analyses were applied to analyze the relationship between different variables and patient prognosis, and a multivariate Cox proportional hazard model was used to estimate correlations between the research variables and the prognosis and to evaluate the hazard ratio (HR) and 95% confidence interval (CI). All analyses were performed using SPSS25.0 software (SPSS Inc, Chicago, IL, USA). The differences of P<0.05 were considered statistically significant.

## Results

### Baseline Characteristics

A total of 525 consecutive LA-NPC patients were treated in the First Affiliated Hospital of Nanchang University from January 2015 to March 2018. According to inclusion criteria, 386 patients were enrolled in this study and subsequently divided into IC<3 (247 patients) and IC≥3 (139 patients) treatment groups based on the numbers of IC cycles they received.

The baseline clinical characteristics of the different groups are shown in **Table 1**. Notably, the IC<3 group had significantly more male patients (*p*=0.041), received more concurrent chemotherapy (CCT) (*p*<0.001), and included more AC patients (*p*<0.001) compared to the IC≥3 group. In addition, more patients who received the TP regimen of IC (*P*<0.001) were found in the IC≥3 group. There were no significant differences found between groups in other clinical characteristics, such as age, clinical stage, toxic reaction, T category, or N category. Furthermore, treatment failure was not significantly different between the IC<3 and IC≥3 groups.

**Table 1:** Basic characteristics of the patients in the IC <3 and IC ≥3 groups.

Characteristics	IC<3	IC≥3	*P-value*	
	n (%)	n (%)		
Total	247 (64.0)	139 (36.0)		
Gender			0.041	
Male	189 (76.5)	93 (66.9)		
Female	58 (23.5)	46 (33.1)		
Age (years old)			0.241	
<50	106 (42.9)	69 (49.6)		
≥50	141 (57.1)	70 (50.4)		
KPS			0.802	
80	114 (46.2)	66 (47.5)		
90	133 (53.8)	73 (52.5)		
Histology^a^			0.261	
WHO I	2 (0.8)	3 (2.2)		
WHO II (IIA+IIB)	245 (99.2)	136 (97.8)		
T category^b^			0.287	
T1-2	133 (53.8)	67 (48.2)		
T3-4	114 (46.2)	72 (51.8)		
N category^b^			0.893	
N0-1	17 (6.9)	10 (7.2)		
N3-4	230 (93.1)	128 (92.8)		
Clinical stage^b^			0.127	
III	175 (70.9)	88 (63.3)		
IVA	72 (29.1)	51 (36.7)		
CCT			<0.001	
Yes	149 (60.3)	55 (39.6)	
No	98 (39.7)	84 (60.4)	
IC regimen			<0.001	
TP	113 (45.7)	87 (62.6)	
DP	100 (40.5)	28 (20.1)	
TPF	23 (9.3)	20 (14.4)	
Other	11 (4.5)	4 (2.9)	
AC			<0.001	
Yes	169 (68.4)	55 (39.6)	
No	78 (31.6)	84 (60.4)	
Toxic reaction^c^			0.819	
0-2	193 (78.1)	109 (79.1)	
3-4	54 (21.9)	29 (20.9)	
Recurrence			
Yes	29	23	0.214	
No	218	116	
Metastasis			0.126	
Yes	40	14	
No	207	125	

KPS, Karnofsky scale; CCT, concurrent chemotherapy; IC, induction chemotherapy; AC, adjuvant chemotherapy; TP, paclitaxel + platinum; DP, docetaxel + platinum; TPF, taxane, platinum, and fluorouracil.

a According to the 2003 edition of the WHO.

b According to the 8th edition of the UICC/AJCC staging system.

c According to the NCI-CTCAE ver. 4.0.

### Survival Analysis

**Figure 2:**
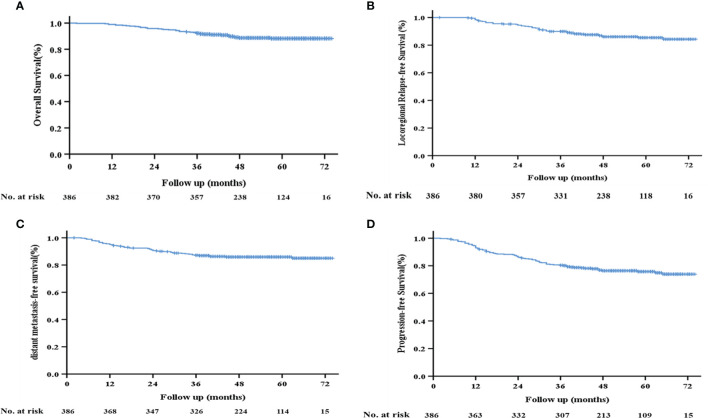
Kaplan–Meier survival curves of the 386 patients with LA-NPC. **(A)** Overall survival; **(B)** locoregional relapse-free survival; **(C)** distant metastasis-free survival; **(D)** progression-free survival.

The median follow-up time in this study was 53 months (range: 2–74 months). The 3-year OS, local recurrence-free survival (LRFS), DMFS, and PFS rates of the 386 patients were 92.2%, 88.9%, 87.2%, and 80.4%, respectively (**Figure 2**).

**Figure 3:**
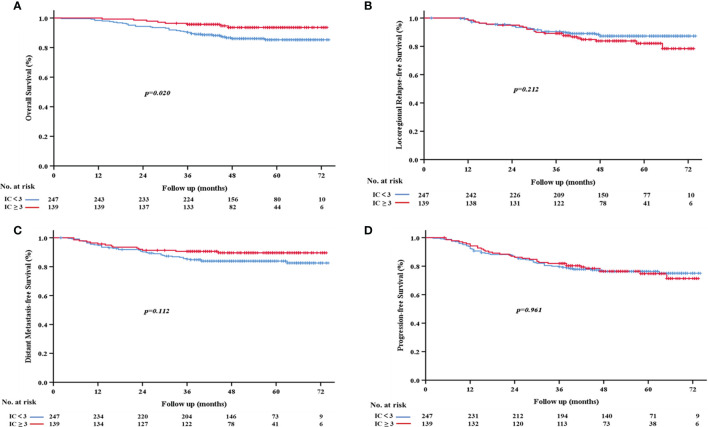
Kaplan-Meier survival curves based on the number of cycles of induction chemotherapy. **(A)** Overall survival; **(B)** locoregional relapse-free survival; **(C)** distant metastasis-free survival; **(D)** progression-free survival.

We also assessed the relationship between the number of IC cycles and survival rates and found that the IC≥3 group had better survival outcomes. Specifically, the 3-year OS rate of the IC≥3 group was significantly higher than that of the IC<3 group (95.7% vs. 90.3%, *p*=0.020; **Figure 3A**). However, no significant differences were observed in 3-year LRFS (*p*=0.212, **Figure 3B**), DMFS (*p*= 0.112, **Figure 3C**), or PFS (*p*=0.961, **Figure 3D**) between the two groups.

### Prognostic Factors

To evaluate whether the IC cycle number was predictive of survival outcomes, we performed the univariate and multivariate analyses of all 386 patients. As shown in **Table 2**, the univariate analysis indicated that the T stage and clinical stage were prognostic factors for OS (*p*=0.026 and *p*=0.017, respectively), LRFS (*p*=0.015 and *p*=0.015, respectively), DMFS (*p*=0.019 and *p*=0.028, respectively), and PFS (*p*=0.011 and *p*=0.001, respectively). However, the results of the multivariate analysis revealed that the clinical stage was only an independent factor for PFS (95% CI 0.343~0.844, HR=0.538, *p*=0.007; **Table 3**).

**Table 2:** Univariate Cox regression analysis for all patients.

Characteristics	OS (%)	*P-value*	LRFS (%)	*P-value*	DMFS (%)	*P-value*	PFS (%)	*P-value*
Gender		0.398		0.296		0.259		0.332
Male	88.3		85.5		84.8		75.1	
Female	91.3		89.4		89.4		79.8	
Age		0.220		0.424		0.438		0.367
≤50	90.9		87.6		87.1		78	
>50	87.5		85.5		85.0		74.9	
T category		0.026		0.015		0.019		0.011
T1-2	92.5		90.5		90.0		81.5	
T3-4	85.5		82.3		81.7		70.8	
N category		0.518		0.064		0.714		0.242
N0-1	85.2		74.1		88.9		66.7	
N3-4	89.4		87.4		85.8		77	
Clinical stage		0.017		0.005		0.028		0.001
III	91.6		89.7		88.6		81.4	
IVA	83.7		79.7		80.5		65.6	
CCT		0.221		0.778		0.202		0.556
Yes	87.3		86.3		83.8		75.4	
No	91.2		86.8		88.5		77.5	
AC		0.369		0.957		0.546		0.362
Yes	90.2		86.2		86.6		77.6	
No	87.7		87.0		85.2		74.7	
IC regimen		0.978		0.806		0.480		0.558
TP	88.5		85.0		88.0		77.0	
DP	89.8		91.4		82.8		76.6	
TPF	93.0		81.4		86.0		73.8	
Other	80.0		80.0		86.7		73.3	
IC cycle		0.024		0.215		0.117		0.961
<3	86.2		88.3		83.8		76.4	
≥3	94.2		83.5		89.9		76.3	

OS, overall survival; LRFS, locoregional relapse-free survival; DMFS, distant metastasis-free survival; PFS, progression-free survival; IC, induction chemotherapy; TP, taxane and platinum; DP, docetaxel and platinum; TPF, taxane, platinum, and fluorouracil.

**Table 3:** Multivariate Cox regression analysis for all patients.

Outcome	Variables		HR		95%CI		*P*-value	
OS	Gender (male vs. female) T category (T1-2 vs. T3-4) Clinical stage (III vs. IVA) AC (yes vs. no) CCT (yes vs. no) IC cycle (IC≥3 vs. IC<3)	1.245		0.594~2.607		0.562	
		0.575		0.293~1.131		0.109	
		0.530		0.273~1.028		0.060	
		0.570		0.306~1.061		0.076	
		1.015		0.526~1.957		0.966	
		0.326		0.145~0.733		0.007	
LRFS	Gender (male vs. female) T category (T1-2 vs. T3-4) Clinical stage (III vs. IVA) AC (yes vs. no) CCT (yes vs. no) IC cycle (IC≥3 vs. IC<3)	1.459		0.747~2.850		0.269	
		0.612		0.335~1.117		0.110	
		0.565		0.313~1.020		0.058	
		1.086		0.605~1.947		0.783	
		0.987		0.557~1.750		0.965	
		1.344		0.736~2.455		0.336	
DMFS	Gender (male vs. female) T category (T1-2 vs. T3-4) Clinical stage (III vs. IVA) AC (yes vs. no) CCT (yes vs. no) IC cycle (IC≥3 vs. IC<3)	1.372		0.706~2.664		0.351	
		0.599		0.332~1.080		0.089	
		0.639		0.356~1.146		0.133	
		0.700		0.402~1.222		0.210	
		1.131		0.637~2.009		0.674	
		0.552		0.289~1.055		0.072	
PFS	Gender (male vs. female) T category (T1-2 vs. T3-4) Clinical stage (III vs. IVA) AC (yes vs. no) CCT (yes vs. no) IC cycle (IC≥3 vs. IC<3)	1.241		0.761~2.026		0.387	
		0.711		0.454~1.113		0.136	
		0.538		0.343~0.844		0.007	
		0.767		0.498~1.182		0.229	
		0.980		0.636~1.509		0.926	
		0.878		0.553~1.393		0.581	

All variables are categorical variables. HRs were calculated for gender (male vs. female), T category (T1-2 vs. T3-4), Clinical stage (III vs. IVA), AC (yes vs. no), CCT (yes vs. no) and IC cycle (IC≥3 vs. IC<3). OS, overall survival; LRFS, locoregional relapse-free survival; DMFS, distant metastasis-free survival; PFS, progression-free survival; IC, induction chemotherapy; CCT, concurrent chemotherapy; AC, adjuvant chemotherapy; CI, confidence interval. HR, hazard radio.

In addition, univariate analysis showed that the IC cycle number significantly affected OS (*p*=0.024) but not LRFS (*p*=0.215), DMFS (*p*=0.117), or PFS (*p*=0.961) (**Table 2**). Similarly, multivariate analysis showed that the number of IC cycles was also an independent prognostic factor for OS (95% CI 0.145~0.733, HR= 0.326, *p*=0.007) but not for LRFS, DMFS, or PFS (**Table 3**).

Both the univariate and multivariate analyses of the potential effects of gender, age, N category, CCT, AC, and IC regimen on survival outcomes indicated that none of these factors were associated with the prognosis (**Tables 2**, **3**). These findings suggested that only the IC cycle was an independent factor for OS and that the clinical stage was an independent factor for PFS.

### Subgroup Analysis

**Figure 4:**
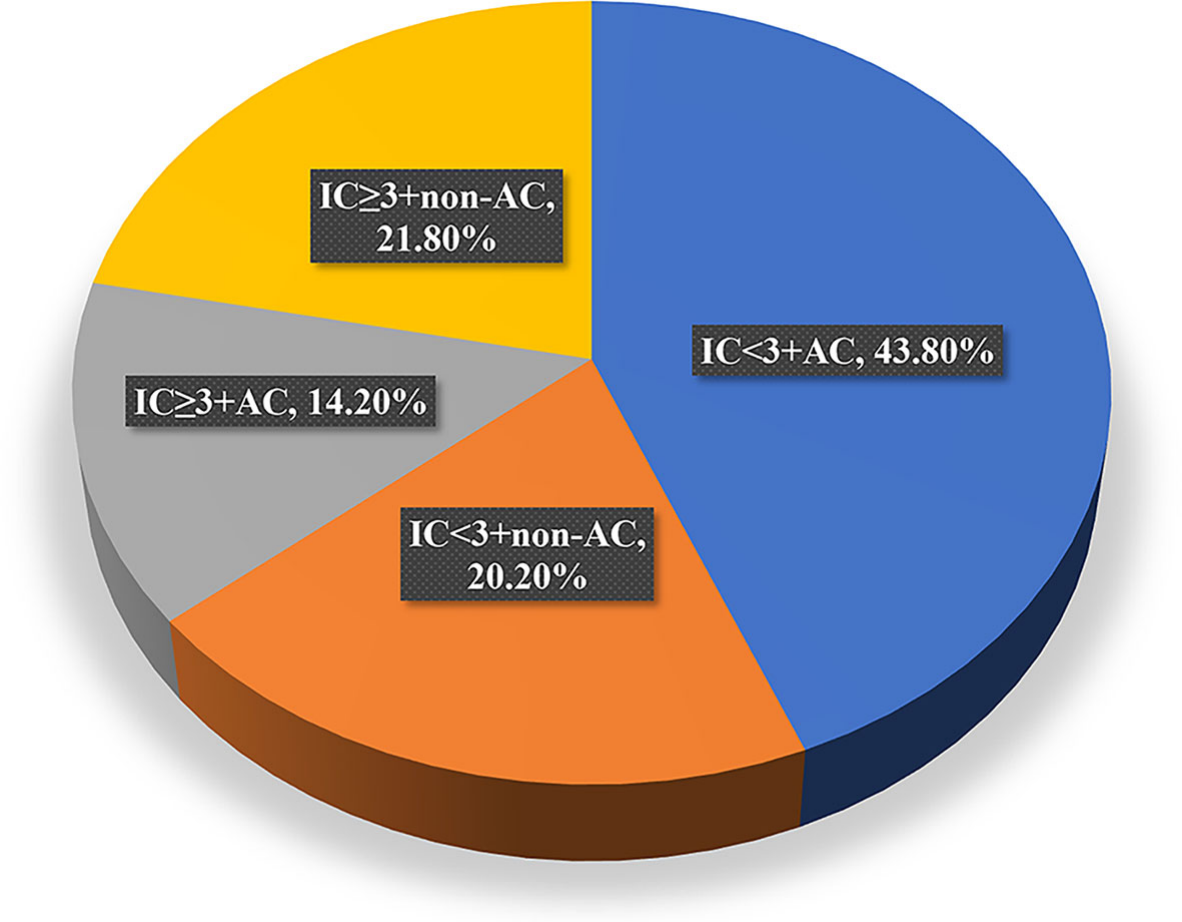
Distribution of patients receiving each of the four treatment modalities. IC, induction chemotherapy; AC, adjuvant chemotherapy.

**Figure 5:**
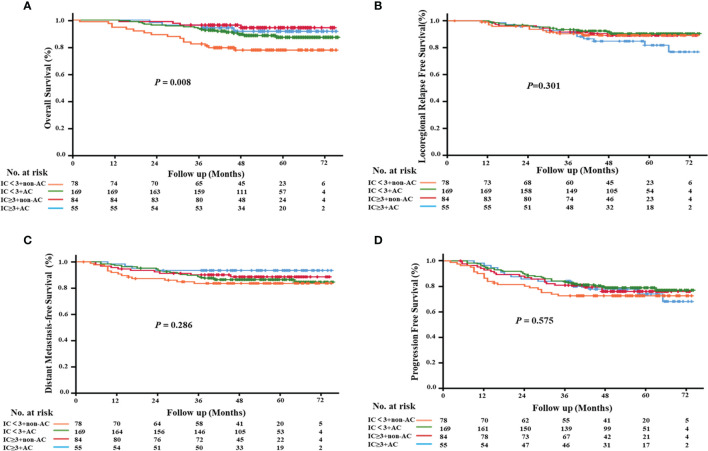
Kaplan–Meier survival curves of the four treatment modalities. **(A)** overall survival; **(B)** locoregional relapse-free survival; **(C)** distant metastasis-free survival; **(D)** progression-free survival.

To evaluate the role of AC in the survival outcomes of patients with locally advanced nasopharyngeal carcinoma, we further subdivided the patient cohort into four groups: IC<3+non-AC (20.2%), IC<3+AC (43.8%), IC≥3+non-AC (21.8%), and IC≥3+AC (14.2%, **Figure 4**). We found that patients in the IC<3+non-AC group had significantly lower OS rates compared to the other three groups (all *p*<0.05; **Figure 5A**). There were no significant differences among the four groups in LRFS, DMFS, and PFS (all *p*>0.05; **Figures 5B–D**, respectively). In particular, patients in the IC≥3+non-AC group had the highest OS rate (HR=0.208, 95% CI 0.069~0.631, *p*=0.006) (**Table 4**) according to the OS curve and multivariate analysis. In addition, we performed an analysis to account for different potential contributing factors and found that gender, T and clinical stages, and CCT did not affect survival outcomes (**Table 4**).

**Table 4:** Multivariate Cox regression analysis of overall survival.

Variables	HR	95% CI	*P-value*
Gender (male/female)	1.303	0.619~2.740	0.486
T category (T0-1/T2-3)	0.571	0.291~1.122	0.104
Clinical stage (III/IVA)	0.548	0.282~1.067	0.077
CCT (yes/no)	1.021	0.282~1.967	0.950
Treatment modalities			0.010
IC≥3+AC	0.285	0.093~0.877	0.029
IC≥3+non-AC	0.208	0.069~0.631	0.006
IC<3+AC	0.468	0.238~0.920	0.028
IC<3+non-AC	Reference	–	–

All variables are categorical variables. HRs were calculated for gender (male vs. female), T category (T1-2 vs. T3-4), Clinical stage (III vs. IVA), AC (yes vs. no), CCT (yes vs. no) and treatment modalities (IC≥3+AC, IC≥3+non-AC, IC<3+AC and IC<3+non-AC). OS, overall survival; IC, induction chemotherapy; CCT, concurrent chemotherapy; AC, adjuvant chemotherapy; CI, confidence interval; HR, hazard radio.

### Treatment Toxicities

The comparison of treatment toxicities between each group showed no significant differences in the incidence of grade 3-4 hematological toxicity (i.e., leukopenia, neutropenia, anemia, and thrombocytopenia) among the four treatment modes. However, sequential AC after IC with CCRT/IMRT can increase the risk of 3-4 leukopenia and neutropenia, although no significant differences were found (*p*=0.761 and *p*=0.402, respectively; **Table 5**).

**Table 5:** The toxic reactions among the four groups.

Toxic reactions (3-4)	IC≥3+AC	IC≥3+nonAC	IC<3+AC	IC<3+nonAC	*P*-value
	N (%)				
Leukopenia	8 (14.5)	12 (14.3)	31 (18.3)	11 (14.1)	0.761
Neutropenia	13 (23.6)	13 (15.5)	37 (21.9)	12 (15.4)	0.402
Anemia	0	1 (1.2)	2 (1.2)	0	N
Thrombocytopenia	1 (1.8)	3 (3.6)	4 (2.4)	2 (2.6)	N

OS, overall survival; IC, induction chemotherapy; AC, adjuvant chemotherapy.

## Discussion

Patients with LA-NPC typically have relatively poor survival outcomes despite augmenting the treatment intensity with CCRT. Currently, a combination of CCRT with IC is used to improve survival outcomes (2); however, the optimal cumulative dose of IC for LA-NPC has remained unclear. In this study, we retrospectively investigated the difference in the impact of the IC cycle number on patient outcomes, as well as the effects of AC in the treatment of LA-NPC.

Consistent with previous studies (13, 18), our results indicated that IC cycles were an independent prognostic factor for the OS rate. Patients who were treated with three or more IC cycles had remarkably higher survival outcomes compared to patients with fewer than three IC cycles. Similarly, Wei et al. found that patients in N2-3 NPC treated with 4 cycles of IC had better OS, DMFS, LRFS, and MFS than those treated with 2 cycles or without IC (15). Wang et al. showed that >2 cycles of IC prior to CCRT resulted in better DMFS and PFS rates in patients with LA-NPC (14). Additionally, Sun et al. demonstrated that three cycles of TPF IC before CCRT significantly improved PFS in patients with LA-NPC (80% vs. 72%, HR=0.68, *P*=0.034) *(* 5). These findings clearly indicate that more than two cycles of IC lead to better patient outcomes.

Other recent studies have proposed that AC following CCRT can also result in improved survival outcomes in LA-NPC patients, although its effects on OS and RFS are still controversial. Clinical trials and meta-analyses have both reported that AC plus CCRT did not significantly improve the survival of patients with stage III–IVB NPC and could even increase the incidence of G3/4 toxicities (17, 19–21). In this study, univariate and multivariate Cox regression analyses suggested that AC was not a significant predictive factor for OS, PFS, LRFS, or DMFS in this cohort. However, it is noteworthy that the addition of AC treatments can lead to remarkably increased 3-year OS rates in patients treated with <3 IC cycles compared to patients who received IC < 3 + non-AC treatment. By contrast, sequential AC after IC plus CCRT/IMRT did not improve survival outcomes in patients receiving ≥3 IC cycles. These findings indicated that sequential adjuvant chemotherapy after CCRT/IMRT may be considered for patients with LA-NPC who received <3 cycles of induction chemotherapy when treatment is tolerated. Additionally, it may be unnecessary to provide AC treatment for LA-NPC patients who received more than 3 cycles of IC.

There were also a few limitations in our study. Firstly, it was a retrospective study from a single treatment center, and the follow-up time was insufficient. Secondly, the data on treatment-related adverse reactions are not adequate. Moreover, although we eliminated selection bias, survival outcomes might be affected by other confounding factors. Thus, long-term follow-up and multicenter prospective trials are needed to confirm the optimal number of IC cycles and the most effective combination of treatments for patients suffering from this severe disease.

## Conclusion

In conclusion, the results of this retrospective study suggest that the number of IC cycles is an independent factor for OS in patients with LA-NPC. More than two cycles of IC treatment plus CCRT/IMRT may also be a promising option for LA-NPC patients, and sequential AC after CCRT/IMRT may be beneficial for patients who received <3 cycles of IC.

## Data Availability Statement

The original contributions presented in the study are included in the article/supplementary material. Further inquiries can be directed to the corresponding authors.

## Ethics Statement

The studies involving human participants were reviewed and approved by the ethics committee of the First Affiliated Hospital of Nanchang University. Written informed consent to participate in this study was provided by the participants’ legal guardian/next of kin.

## Author Contributions

SL, YD, and QL contributed to carrying out the research. ZX, PZ, CZ, and WD analyzed data and prepared the manuscript. WZ and XZ designed the study and supervised the experiments. SL and YD edited the manuscript. YY revised the manuscript. All authors contributed to the article and approved the submitted version.

## Funding

This study was supported by the National Natural Science Foundation of China (No. 82060424 and 81260345).

## Conflict of Interest

The authors declare that the research was conducted in the absence of any commercial or financial relationships that could be construed as a potential conflict of interest.

## Publisher’s Note

All claims expressed in this article are solely those of the authors and do not necessarily represent those of their affiliated organizations, or those of the publisher, the editors and the reviewers. Any product that may be evaluated in this article, or claim that may be made by its manufacturer, is not guaranteed or endorsed by the publisher.
